# Transcript length bias in RNA-seq data confounds systems biology

**DOI:** 10.1186/1745-6150-4-14

**Published:** 2009-04-16

**Authors:** Alicia Oshlack, Matthew J Wakefield

**Affiliations:** 1 Bioinformatics Division, Walter and Eliza Hall Institute of Medical Research, Parkville, Vic 3052, Australia

## Abstract

**Background:**

Several recent studies have demonstrated the effectiveness of deep sequencing for transcriptome analysis (RNA-seq) in mammals. As RNA-seq becomes more affordable, whole genome transcriptional profiling is likely to become the platform of choice for species with good genomic sequences. As yet, a rigorous analysis methodology has not been developed and we are still in the stages of exploring the features of the data.

**Results:**

We investigated the effect of transcript length bias in RNA-seq data using three different published data sets. For standard analyses using aggregated tag counts for each gene, the ability to call differentially expressed genes between samples is strongly associated with the length of the transcript.

**Conclusion:**

Transcript length bias for calling differentially expressed genes is a general feature of current protocols for RNA-seq technology. This has implications for the ranking of differentially expressed genes, and in particular may introduce bias in gene set testing for pathway analysis and other multi-gene systems biology analyses.

**Reviewers:**

This article was reviewed by Rohan Williams (nominated by Gavin Huttley), Nicole Cloonan (nominated by Mark Ragan) and James Bullard (nominated by Sandrine Dudoit).

## Background

High throughput sequencing is likely to become the platform of choice for transcriptome analysis. The ability of sequencing platforms to interrogate the whole transcriptional landscape provides new insights into the levels of transcriptional complexity in biological systems. Transcript sequencing also provides novel opportunities such as quantitatively measuring splicing variants [1] and single nucleotide polymorphisms (SNPs) for allele specific expression without any prior knowledge. However this new level of detail needs careful statistical modeling to provide the promised benefits of the new technology.

Different technologies display different data features and will therefore have different strengths and weaknesses. Investigation of the technical and statistical attributes of the data will reveal the advantages and disadvantages of each technology. We hypothesize, that using statistical methods to detect differential expression between samples is biased by transcript length and that this bias is inherent to the standard RNA-seq process.

Current RNA-seq protocols use an mRNA fragmentation approach prior to sequencing to gain sequence coverage of the whole transcript. This means, in simple terms, that the total number of reads for a given transcript is proportional to the expression level of the transcript multiplied by the length of the transcript. In other words a long transcript will have more reads mapping to it compared to a short gene of similar expression. Since the power of an experiment is proportional to the sampling size, there is more power to detect differential expression for longer genes. This is an inherent property of the data and will not be altered by any process involving sequencing of full length transcripts with fragments shorter than the transcript. By contrast, intensity measurements from microarrays are proportional only to the expression level of the transcript plus any features intrinsic to the probe itself such as GC content [2,3]. The feature of higher sampling for longer transcripts in RNA-seq data becomes important in situations looking at identifying differentially expressed genes between samples. Most statistical methods for detection of differential expression will have more power for transcripts with a larger number of reads. Short transcripts will therefore always be at a statistical disadvantage relative to long transcripts in the same sample.

Here we explore several previously published datasets of high throughput RNA sequencing and show that the most widely used protocols for RNA-seq can detect more differential expression in longer transcripts compared to shorter ones. This bias does not exist for microarray platforms, which uses a single or set of diagnostic probes to assess expression levels. We also demonstrate that the inherent biases presented here are shown to exist in all experiments analyzed so far independent of the specific samples, platforms or statistical analysis.

## Results

Based on a simplified calculation, we can show that the ability to detect differential expression using a very simple testing procedure depends on the length of the transcript (see methods). To empirically investigate the effect of transcript length bias in RNA-seq and microarrays we used three different published data sets that sequence full length transcripts. The first data set compares sequencing from the Illumina Genome Analyzer with Illumina microarrays and looks for differential expression between a human embryonic kidney and B cell line [4]. The second uses SOLiD sequencing to compare mouse embryonic stem cells and embryoid bodies [5] and the third compares Illumina sequencing with Affymetrix microarrays in a human liver and kidney sample [6]. In order to determine which genes are differentially expressed, each study uses a different statistical approach to calculate significance. Here we use these statistics to examine the behavior of differential expression with transcript length.

**Figure 1:**
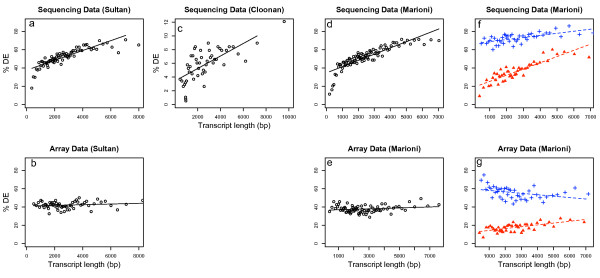
**Differential expression as a function of transcript length**. The data is binned according to transcript length and the percentage of transcripts called differentially expressed using a statistical cut-off is plotted (points). A linear regression is also plotted (lines). **a **– **e **use all the data from RNA-seq and the microarrays from studies [4-6] respectively. **f **and **g **plot 33% of genes with highest expression levels (blue crosses) and 33% of genes with low expression (red triangles) taken from the microarray data for genes which appear on both platforms in [6]. The regression gives a significant trend for the percent of differential expression with transcript length for **a**, **c**, **d **and **f **and the lowly expressed genes in **g**. Note that this figure illustrates common data features between disparate experiments and is not a comparison between platforms, methods or experiments.

For each platform we first binned all genes into equal gene number bins based on their transcript length. Next we designated genes as differentially expressed (DE) based on a cut-off from the statistical procedure defined in the relevant publication. We found the specific statistical cut-off used made no qualitative difference to the results. We then calculated the percentage of DE genes for each bin. Figure 1 shows the percentage of DE genes plotted as a function of transcript length for both RNA-seq and microarrays for each experiment. Clearly the ability to detect DE is strongly associated with transcript length for RNA-seq regardless of the platform, statistical analysis procedure or overall proportion of differential expression (Figure 1a,c and 1d). As expected no such trend is observed for the microarray data from two different platforms (Figure 1b and 1e).

To further investigate the transcript length bias we used data from Marioni et al (2008) and looked at transcripts that appear in both the RNA-seq and microarray data. We divided the genes into three equal groups based on the average intensity level measured on the microarrays. We then calculated %DE as a function of length for the high and low expression groups (Figure 1f and 1g). RNA-seq shows an even stronger length bias for lowly expressed genes, which is somewhat ameliorated, but still significant, for highly expressed genes. We believe the slope is lower in highly expressed genes because nearly all of these genes have enough power to be called differentially expressed in this data set even though the p-values are higher for shorter genes. By contrast, no significant trend is observed for highly expressed genes using the microarray platform and only a slight trend is induced for genes with low expression. Although the overall number of DE genes called may be larger for RNA-seq, increasing the number of replicates would increase the number of DE genes detected by the microarrays. Careful calibration of the absolute rates of DE between platforms could be the subject of a further investigation, however the trends identified here scale with, and are robust to, the specific statistical cut-off used.

**Figure 2:**
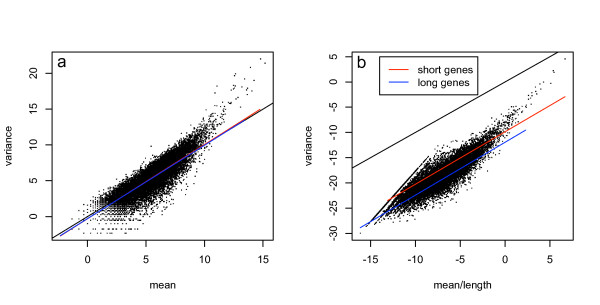
**Mean-variance relationship**. Here we show the sample variance across lanes in the liver sample from the Marioni et al[6] data plotted as a function of the mean for each gene (a). Next we have the same data where the tag counts for each gene are divided by the length of the gene (b). The red line fits a linear relationship between the mean and variance for the one third of shortest genes while the blue line is the linear fit to the longest genes. In plot **a **the fits are very close to the line of equality between mean and variance (black line) which is what would be expected from a Poisson process. In plot **b **the short genes have higher variance for a given expression level than long genes.

Many of the current statistical methods use a measure of expression level normalized by the length of the gene. This gives an unbiased measure of the expression level but also affects the variance of the data in a length dependent manner resulting in the same bias to differential expression estimation (see methods for a toy example). To demonstrate this effect we show the sample mean and variance of each gene calculated from the replicate lanes of the Marioni et al. data. In figure 2a we show that the sample mean and variance are approximately equal as would be expected for a Poisson random variable. However, when the mean is divided by the length of the transcript the relationship becomes more complex and the data is no longer Poisson. Figure 2b shows the same data with the tag counts for each transcript divided by the length of the transcript. The two fitted curves show the mean-variance relationship for one third of the data with the longest transcripts and with the shortest transcripts. It is easy to see that for genes normalized by length shorter transcripts have larger variance for the same expression level compared to longer transcripts.

**Figure 3:**
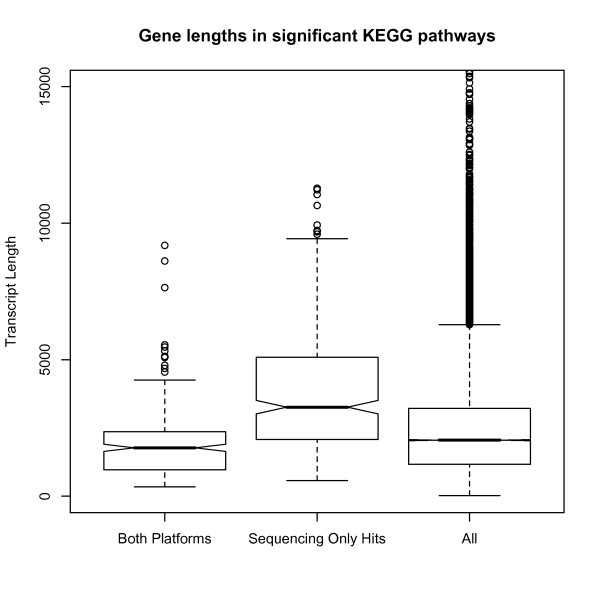
**Length of genes found in KEGG pathways significantly over represented with differentially expressed genes**. The first box in the plot represents the length of genes found in the four significant categories from both platforms. The second box is the length of genes found in categories significant only in the sequencing data. The third box is the length of all genes in common to both technologies. It can be seen that categories unique to the sequencing data tend to have longer transcripts.

The consequences of transcript length bias in RNA-seq data becomes most problematic when comparing between genes or sets of genes with different lengths. This is most likely to occur when doing gene set testing in systems biology, where specific gene sets have a length bias compared to other sets of genes. If a set contains genes shorter than average it will appear under-represented in differential expression whereas if the set contains genes longer than average the category is more likely to be over-represent in differential expression. To demonstrate this effect we looked for over-represented KEGG pathways using the Marioni et al RNA-seq and microarray data. In each analysis we only used genes found on both platforms and then performed a pathway analysis using the DAVID software [7,8]. We found several pathways which were over-represented for differential expression between liver and kidney. Tables 1 and 2 show all of the pathways over-represented below a p-value of 0.1 (however after multiple testing correction only the top 16 categories remain in the microarray data at the same significance as the sequencing data). Categories highlighted in bold do not appear overrepresented anywhere in the list from the other platform. After multiple testing correction the microarray platform contains four pathways below a threshold of 0.1 all of which are found in the sequencing data. By contrast the RNA-seq data contains nine categories of which three are not contained anywhere on the array data. Figure 3 shows the lengths of the genes associated with each of these categories. The first box gives the distribution of genes in pathways appearing significant on both platforms. The second box gives genes appearing significant only in the sequencing platform (i.e. do not appear anywhere on the list from the array platform) and the third box is the length distribution of all the transcripts in the analysis. It can be clearly seen that genes in categories only over-represented on the RNA-seq platform are significantly longer than average.

**Table 1:** Overrepresented KEGG pathways using microarrays.

Term	Count	Pop Hits	PValue	Benjamini
hsa04610:Complement and coagulation cascades	54	68	2.36E-10	5.44E-08
hsa00980:Metabolism of xenobiotics by cytochrome P450	45	65	6.97E-06	5.37E-04
hsa00190:Oxidative phosphorylation	74	121	5.83E-06	6.73E-04
hsa00120:Bile acid biosynthesis	25	36	0.00126	0.0702

hsa00260:Glycine, serine and threonine metabolism	29	45	0.00246	0.107
hsa00591:Linoleic acid metabolism	20	31	0.01496	0.252
hsa00380:Tryptophan metabolism	35	60	0.00764	0.255
hsa05010:Alzheimer's disease	19	29	0.0149	0.271
hsa00363:Bisphenol A degradation	11	14	0.0188	0.287
hsa00020:Citrate cycle (TCA cycle)	18	27	0.0148	0.291
hsa04514:Cell adhesion molecules (CAMs)	65	126	0.0108	0.300
**hsa00040:Pentose and glucuronate interconversions**	**16**	**23**	**0.0141**	**0.305**
hsa03320:PPAR signaling pathway	39	70	0.0125	0.305
hsa00650:Butanoate metabolism	26	45	0.0280	0.374
hsa00280:Valine, leucine and isoleucine degradation	25	44	0.03995	0.425
**hsa00361:gamma-Hexachlorocyclohexane degradation**	**15**	**23**	**0.0379**	**0.428**
hsa00903:Limonene and pinene degradation	18	29	0.0360	0.432
hsa00230:Purine metabolism	69	143	0.0472	0.462
hsa00071:Fatty acid metabolism	25	45	0.0536	0.488
**hsa00670:One carbon pool by folate**	**11**	**16**	**0.0622**	**0.524**
hsa00620:Pyruvate metabolism	23	42	0.0776	0.526
**hsa00910:Nitrogen metabolism**	**14**	**23**	**0.0874**	**0.529**
hsa00010:Glycolysis/Gluconeogenesis	31	59	0.0666	0.531
**hsa04330:Notch signaling pathway**	**25**	**46**	**0.0703**	**0.535**
hsa00860:Porphyrin and chlorophyll metabolism	21	38	0.0857	0.535
**hsa02010:ABC transporters – General**	**24**	**44**	**0.0738**	**0.537**
**hsa00150:Androgen and estrogen metabolism**	**28**	**53**	**0.0774**	**0.539**
hsa00410:beta-Alanine metabolism	15	25	0.0838	0.541
hsa00052:Galactose metabolism	18	32	0.0996	0.554
**hsa04614:Renin-angiotensin system**	**11**	**17**	**0.0977**	**0.559**

Categories in bold are not found to be overrepresented in the RNA-seq data.

**Table 2:** Over represented KEGG pathways using Illumina sequencing.

Term	Count	Pop Hits	PValue	Benjamini
hsa04610:Complement and coagulation cascades	60	68	3.08E-08	7.11E-06
**hsa04910:Insulin signaling pathway**	**96**	**133**	**1.06E-04**	**0.0122**
hsa00020:Citrate cycle (TCA cycle)	25	27	2.23E-04	0.0170
hsa00120:Bile acid biosynthesis	31	36	4.23E-04	0.0242
hsa00071:Fatty acid metabolism	37	45	5.35E-04	0.0244
hsa00980:Metabolism of xenobiotics by cytochrome P450	50	65	7.29E-04	0.0277
hsa00190:Oxidative phosphorylation	85	121	0.001155	0.0374
**hsa00310:Lysine degradation**	**38**	**48**	**0.001627**	**0.0459**
**hsa04510:Focal adhesion**	**128**	**196**	**0.004824**	**0.0966**

**hsa00051:Fructose and mannose metabolism**	**33**	**42**	**0.00463**	**0.102**
hsa00650:Butanoate metabolism	35	45	0.00448	0.109
**hsa04520:Adherens junction**	**52**	**74**	**0.0129**	**0.171**
**hsa04810:Regulation of actin cytoskeleton**	**133**	**208**	**0.0116**	**0.175**
**hsa04912:GnRH signaling pathway**	**64**	**93**	**0.0109**	**0.177**
hsa00010:Glycolysis/Gluconeogenesis	43	59	0.0110	0.177
hsa00230:Purine metabolism	94	143	0.0128	0.180
hsa00280:Valine, leucine and isoleucine degradation	33	44	0.0147	0.183
hsa05010:Alzheimer's disease	23	29	0.0200	0.228
hsa00620:Pyruvate metabolism	31	42	0.0262	0.253
**hsa05210:Colorectal cancer**	**57**	**84**	**0.0250**	**0.254**
hsa00260:Glycine, serine and threonine metabolism	33	45	0.0241	0.256
hsa04514:Cell adhesion molecules (CAMs)	82	126	0.0285	0.262
**hsa04670:Leukocyte transendothelial migration**	**74**	**113**	**0.0315**	**0.275**
hsa00220:Urea cycle and metabolism of amino groups	23	30	0.0359	0.297
**hsa04360:Axon guidance**	**81**	**126**	**0.0429**	**0.313**
**hsa04370:VEGF signaling pathway**	**47**	**69**	**0.0401**	**0.315**
**hsa05120:Epithelial cell signaling in Helicobacter pylori infection**	**47**	**69**	**0.0401**	**0.315**
hsa00052:Galactose metabolism	24	32	0.0448	0.315
**hsa00480:Glutathione metabolism**	**27**	**37**	**0.0495**	**0.315**
hsa00903:Limonene and pinene degradation	22	29	0.0488	0.319
**hsa05040:Huntington's disease**	**22**	**29**	**0.0488**	**0.319**
hsa03320:PPAR signaling pathway	47	70	0.0549	0.327
hsa00380:Tryptophan metabolism	41	60	0.0535	0.328
**hsa05211:Renal cell carcinoma**	**45**	**67**	**0.0605**	**0.330**
hsa00591:Linoleic acid metabolism	23	31	0.0596	0.333
**hsa01510:Neurodegenerative Diseases**	**28**	**39**	**0.0585**	**0.336**
hsa00410:beta-Alanine metabolism	19	25	0.0717	0.356
hsa00363:Bisphenol A degradation	12	14	0.0691	0.360
**hsa00640:Propanoate metabolism**	**24**	**33**	**0.0712**	**0.361**
hsa00860:Porphyrin and chlorophyll metabolism	27	38	0.0752	0.363
**hsa00740:Riboflavin metabolism**	**13**	**16**	**0.0932**	**0.424**
**sa00770:Pantothenate and CoA biosynthesis**	**13**	**16**	**0.0932**	**0.424**
**hsa04530:Tight junction**	**81**	**130**	**0.0991**	**0.429**

Categories in bold are not found to be over represented in the microarray data.

## Discussion

Transcript length bias in RNA-seq data is a predictable consequence of the sampling process and cannot be corrected by dividing by length of the transcript (e.g. the statistical methods in Cloonan et al (2008) or Sultan et al. (2008)). Length bias is expected for a Poisson random variable, where the expected read count of a gene is proportional to the length as well as expression level of a transcript. In other words, the sampling is higher for long genes compared to short genes and therefore there is more power to detect differential expression at a given statistical significance regardless of the specific test used. Statistical tests to detect differential expression between samples require estimates of the mean and the variance of the samples. Dividing the mean by the length of the transcript removes the bias from that measure but subsequently introduces a length bias into the variance and the problem still persists. Similarly, as with microarrays, there is more power to detect differential expression for genes with higher expression levels. However, we have not focused on this phenomenon here as we feel that expression level is a biologically meaningful quantity compared to transcript length bias, which is technical in nature.

Different processing methods can be used with high-throughput sequencing to determine the levels of transcription such as massively parallel signature sequencing (MPSS), serial analysis of gene expression (SAGE), cap analysis of gene expression (CAGE). These methods only count one sequence per transcript hence will not suffer from transcript length effects. However, in many cases researchers will want to examine the full complexity of the transcriptome by sequencing the entire RNA repertoire so we speculate that these unbiased methods will make up a small fraction of RNA-seq experiments. As fragment size is the basis of transcript length bias rather than the number of bases read, improvements in read length on the current platforms will not alter transcript length bias.

Using exon level analysis as a way to reduce the range in transcript lengths may not reduce the bias significantly. Although the lengths of genes are obviously longer than exons when looking at the length of genes and exons in the human genome the interquartile range (IQR) in log_2 _length is similar (genes IQR = 1.45, exons IQR = 1.23). This implies that the number of genes doubling in length is similar to the number of exons doubling in length meaning the bias is just as strong. Extending the sequencing depth will increase the ability to detect differential expression. However as transcript length bias is a relative measure between genes it will not affect the presented results.

Currently the only method we suggest to account for length bias between genes would be to use a fixed length window approach, with a window size smaller than the smallest gene. In this method aggregated tag counts for each window could be calculated and assessed for differential expression. A further extension could combine multiple windows per transcript into a single measure in a way similar to combining multiple probes in the RMA microarray algorithm [9] but this suggestion requires further exploration and analysis. Nonetheless, as analysis needs to be done at the window level this would require discarding some proportion of the data or introducing a variable number of windows per gene. Additionally the small size of the windows will require a larger total number of reads per sample to achieve statistical significance reducing power to the equivalent level of small genes in a gene focused analysis.

It is important to understand that using a statistical cut-off to generate a list of DE genes from aggregated tag counts inevitably will be more sensitive to genes with longer transcripts. Hence gene sets and ontology classes containing genes with different length distributions may look spuriously under or over represented in gene set testing. As gene set testing is an integral part of many systems biology experiments and large biomedical projects such as the International Cancer Genome Consortium, the length bias will significantly impact many applications where RNA-seq is currently being utilized. Sophisticated statistical methodology will be required to develop a new analysis framework that gives similar false discovery rates for different transcript lengths.

## Conclusion

The different strengths of the currently available RNA-seq and microarray technologies make these platforms complementary for comprehensive analysis of the transcriptome. A technical feature of using high-throughput sequencing to interrogate full length transcripts is that longer transcripts produce more reads relative to short transcripts of similar expression. This higher sampling means that there is more statistical power to detect differential expression for long transcripts compared to short ones. Using three published RNA-seq data sets we have demonstrated that, as we hypothesized, longer transcripts have more power to detect differential expression. These data sets each use different samples, platforms and statistical methods. This bias does not exist in microarray data. If unaccounted for, transcript length bias in the ability to detect differential expression can confound system biology and gene set testing approaches. Understanding technical issues in new technologies will lead to the development of more sophisticated analysis methodologies.

## Methods

Here we show, under some very simple assumptions, that when testing for differences between two samples for a given expression level, a longer gene will be more significant that a shorter gene. Let *X *be the measured number of reads in a library mapping to a specific transcript. The expected value of *X *is proportional to the total number of transcripts *N *times the length of the gene *L*



where c is a proportionality constant. Assuming the data is distributed as a Poisson random variable, the variance is equal to the mean.



As an example, we could test if the difference in counts from a particular gene between two samples of the same library size is significantly different from zero using a t-test

(1)

where *D *is the difference in the observed means from two samples and *S.E.(D) *is the standard error of *D*.



The power of the t-test depends on *E(D)/S.E.(D) = δ *which is essentially the non-centrality parameter of the t-distribution



It can be seen that this is proportional to the square root of the length. Therefore for a given expression level the test becomes more significant for longer transcript lengths.

### Dividing by gene length

Given the simple set-up above we can see the effect of dividing expression levels by the length of the gene. Here



and



Given that we assumed that *X *was distributed as Poisson random variable we can see that once we divide by length the distribution is no longer Poisson and *μ*' ≠ *Var*(*μ*').

Using the same format of the t-test above we see that we recover the same length dependence as in equation 1.



where



Again the power of the t-test depends on *E(D)/S.E.(D) = δ *where



We end up with the same test result as equation 1 which still has a square root *L *dependence.

### Empirical data

Processed data was downloaded for each of the three studies presented. Each data set contained a gene ID and the results of a statistical test for differential expression between samples. Each data set uses a different statistical test. Briefly: Marioni et al. [6] modeled their 32 bp reads from the Illumina Genome Analyzer using a Poisson model based on the aggregated tag counts for each gene. A likelihood ratio test was then used to test for significant differences between samples. Cloonan et al. [5] generated tags of 25–35 bp from a SOLiD sequencing machine. The aggregated tag counts for each transcript were divided by the length of the transcript. The data was then quantile normalized and log_2_-transformed. Differential expression was then assessed using an empirical Bayes moderated t-test [10]. Illumina sequencing data of 27 bp read length was generated by Sultan et al. [4] Aggregated tag counts for each gene were divided by the number of unique 27-mers found in the transcript. They then used a method based on the proportion of counts from each library proposed by Audic and Claverie [11] to determine the significant of differential expression.

Transcript lengths for all human transcripts and mouse transcripts were downloaded using BioMart and the length of a gene was calculated as the median length of all transcripts relating to that gene. Each gene was then defined to be either differentially expressed or not differentially expressed based on an arbitrary statistical cut-off. Varying this cut-off made no qualitative difference to the results. For the data from Marioni et al (2008) genes were matched between the RNA-seq platform and the microarray platform. These genes were then divided into three equally sized groups based on the average log_2 _expression level of the six microarrays in their study. The high expression and low expression groups were used in figures 1f and 1g.

## Abbreviations

DE: Differential expression; IQR: interquartile range.

## Competing interests

The authors declare that they have no competing interests.

## Authors' contributions

AO and MJW conceived of the idea and wrote the paper. AO analyzed the data.

## Reviewers' comments

### Reviewers report 1

#### Rohan Williams, John Curtin School of Medical Research, Australian National University, Australia. Nominated by Gavin Huttley

RNA-Seq and related high-throughput sequencing are receiving intense attention due to their potential to survey the transcriptome in an unbiased, global fashion. While it is likely that these sequencing based approaches will permit a major advance on microarray based technologies, it is also highly likely that unanticipated systematic errors will be present in these data and will need to be corrected in order to permit appropriate application. While expression microarrays and tiling arrays are known to be subject to a number of such effects, to date there has been little investigation of issues in the emerging RNA-Seq literature. Oshlack and Wakefield now present a re-analysis of data from several recent RNA-Seq studies to show that identification of differential expression is positively biased towards longer transcripts (and has the potential to impact downstream interpretation at a functional level). Although it is recognised that tag count will be proportional to the product of expression level and transcript length, adjusting for transcript length does not remove this effect: the authors show the effect arises from increased variance for shorted transcripts. They further argue that this effect is unlikely to be removed by exon-level analysis. Interestingly, this effect is not observable in microarray expression platforms. This paper represents an important contribution to the ongoing development of analysis methodology for RNA-Seq and I recommend it for publication in Biology Direct.

### Reviewers report 2

#### Nicole Cloonan, Institute for Molecular Bioscience, The University of Queensland, Australia. Nominated by Mark Ragan

In this paper, the authors describe "transcript length bias" in RNAseq data, which is the reduced statistical power to detect differential gene expression of short mRNAs when compared to long mRNAs using a "shotgun sequencing" approach. As randomly fragmented mRNA molecules will generate less short-read tags for a short transcript than for a longer transcript, changes in expression between two (relatively) poorly sampled transcripts are less discernible from sampling noise. The authors examine three published shotgun sequencing-based studies to show this bias exists in the sequencing data, but not in the corresponding microarray data from the same samples. This bias against short transcripts could lead to a general under-representation in gene set testing for functional categories enriched in short genes (such as cell-cell communication, innate immunity, and signal transduction). This is an important finding that the RNA sequencing community needs to be aware of.

The manuscript is generally well written, and the authors have done well to create a manuscript understandable to a biological audience without specialized mathematical or statistical training. As all of my (generally minor) concerns with this manuscript have been adequately addressed, I recommend this manuscript for publication.

### Reviewers Report 3

#### James Bullard, Division of Biostatistics, School of Public Health, University of California, Berkeley, USA. Nominated by Sandrine Dudoit

In Oshlack and Wakefield the authors demonstrate a relationship between gene-length and observed significance of a statistical test in three published studies (Marioni et al., Cloonan et al., Sultan et al.). The authors demonstrate that this observed tendency is not present in the analysis of the same samples in the Marioni study when microarrays are used. This "bias" is due to the dependence of the variance on the intensity of the read-process which is proportional to the length of the transcript sequenced.

The reviewer recommends the article for publication as the issues presented are both relevantand important. In particular, the issues presented are quite pertinent with the advent of numerous high-throughput sequencing studies. The reviewer believes that in its current form the article would benefit from some revisions to either more rigorously present the mathematics or simply present the statistics described in the offending studies.

Background: paragraph 2, "We hypothesize ..." Why are you hypothesizing? I think that this sentence needs reference to a particular test-statistic, then you really don't need to hypothesize anything.

*Author's response: We believe the statement in the article relates to all statistical analysis methods under the assumptions we have stated however we have not and really cannot test all possible methods. Therefore we have used the word hypothesize but we have also given an example in the methods section*.

Background: paragraph 3, "All methods for detection of ..." Doesn't this sentence appear a bit strong?

Author's response: We amended this to "Most statistical methods..."

Results: paragraph 2, Can you comment why the "length bias" is stronger for more lowly expressed genes? Also, I think it is better to present all of the data on the plots, rather than excluding the middle bin.

Author's response: We have added the sentence: "We believe the slope is lower in highly expressed genes due to the observation that nearly all of these genes have enough power to be called differentially expressed in this data set even though the p-values are higher for shorter genes."

Results: paragraph 3, In the mean-variance plots how do you compute the variance? Is this just the sample variance? What about the different numbers of counts across lanes? As for panel (2), After we divide by length we don't have a Poisson so the mean-variance plot is not correct or at least the proper interpretation of it is non-obvious (isn't it obvious that we will cause a shift on the plot because we are now scaling by length squared?)

Author's response: Yes this is exactly the point we are trying to make. This plot is meant to be more heuristic in nature rather than any rigorous proof that dividing by length doesn't remove the length bias. Therefore we have just used the sample variance without taking into account the different number of counts across lanes as a visual demonstration. To clarify we have also added the sentence: "However, when the mean is divided by the length of the transcript the relationship becomes more complex and the data is obviously no longer Poisson"

Results: paragraph 4, A potentially "better" plot would be boxplots (of gene-length) ordered from largest to smallest KEGG p-value; both for microarray and sequencing data.

*Author's response: Thank you for the suggestion. We felt that the plot you suggested was a little bit more tricky to interpret*.

Methods: paragraph 1, The math is a little sloppy. In general, there is confusion between random variables and parameters. Specifically, I note two obvious errors: 1.) *t *is defined to be one thing (random variables on the rhs of equation (1)) and then redefined to be another thing (parameters on rhs of following definition). 2.) Methods: paragraph 2, *μ' *is a parameter then you do the *Var(μ') *which is incorrect, you probably want to dene an *X' *instead, then you can take variances.

*Author's response: Thanks for pointing this out. We have modified and tidied up the math*.

From your treatment it appears that I can just divide t by √ L to remove the dependence on L in the test-statistic is this correct?

*Author's response: No, I don't think this is possible. A t-test is like a signal to noise ratio and therefore has a specific relationship between the estimate of the mean and the standard error of the estimate. I don't believe this should be broken by essentially dividing the estimate of the mean by √ L*.

## References

[B1] Wang ET, Sandberg R, Luo S, Khrebtukova I, Zhang L, Mayr C, Kingsmore SF, Schroth GP, Burge CB (2008). Alternative isoform regulation in human tissue transcriptomes. Nature.

[B2] Dunning MJ, Barbosa-Morais NL, Lynch AG, Tavare S, Ritchie ME (2008). Statistical issues in the analysis of Illumina data. BMC Bioinformatics.

[B3] Wu Z, Irizarry RA (2005). Stochastic models inspired by hybridization theory for short oligonucleotide arrays. J Comput Biol.

[B4] Sultan M, Schulz MH, Richard H, Magen A, Klingenhoff A, Scherf M, Seifert M, Borodina T, Soldatov A, Parkhomchuk D (2008). A global view of gene activity and alternative splicing by deep sequencing of the human transcriptome. Science.

[B5] Cloonan N, Forrest AR, Kolle G, Gardiner BB, Faulkner GJ, Brown MK, Taylor DF, Steptoe AL, Wani S, Bethel G (2008). Stem cell transcriptome profiling via massive-scale mRNA sequencing. Nat Methods.

[B6] Marioni JC, Mason CE, Mane SM, Stephens M, Gilad Y (2008). RNA-seq: An assessment of technical reproducibility and comparison with gene expression arrays. Genome Res.

[B7] Huang da W, Sherman BT, Lempicki RA (2009). Systematic and integrative analysis of large gene lists using DAVID bioinformatics resources. Nat Protoc.

[B8] Huang da W, Sherman BT, Lempicki RA (2009). Bioinformatics enrichment tools: paths toward the comprehensive functional analysis of large gene lists. Nucleic Acids Res.

[B9] Irizarry RA, Hobbs B, Collin F, Beazer-Barclay YD, Antonellis KJ, Scherf U, Speed TP (2003). Exploration, normalization, and summaries of high density oligonucleotide array probe level data. Biostatistics.

[B10] Smyth GK (2004). Linear models and empirical bayes methods for assessing differential expression in microarray experiments. Stat Appl Genet Mol Biol.

[B11] Audic S, Claverie JM (1997). The significance of digital gene expression profiles. Genome Res.

